# Do Animals Talk?

**Published:** 1892-06

**Authors:** 


### DO ANIMALS TALK?

In the room where the monkeys are kept by a dealer in Washington, there is a cage containing a young white-faced cebus of more than average intelligence, writes R. L. Garner, in the Forum. On the same shelf and in an adjacent cage is the little capuchin Puck. They can easily see and hear each other through the open wire partition which separates them, there being no other obstruction. I have visited Puck for many weeks almost daily, and always supply him with food after requiring him to ask me for it in his own language. Having but little interest in the white-face, who is very shy of me, I rarely showed him the slightest attention until within the past few weeks, when I observed him trying to utter the capuchin sound for food, which always secured for Puck a banana or some nuts. Seeing that Puck was always rewarded for uttering this sound, the little white-face began to try it, and as soon as I discovered his purpose I began to reward him in the same way, and have thus seen one step taken by a monkey in the mastery of another tongue. At first his effort was quite poor and I could not at once decide what he meant; but practice has developed in him great proficiency, and now he speaks it almost as plainly as the capuchin himself. This was doubly interesting to me in view of the fact that I had long believed that no monkey ever acquired the sounds of another species. I frankly admit that this one instance is alone sufficient to cause me to recede from a conclusion rendered untenable by such certain proof, the cogency of which is emphasized by the short time in which it has been accomplished; but I still regard it as a rule that monkeys do not do so.

